# Comparison of ultrasound imaging and cone-beam computed tomography for examination of the alveolar bone level: A systematic review

**DOI:** 10.1371/journal.pone.0200596

**Published:** 2018-10-03

**Authors:** Kim-Cuong T. Nguyen, Camila Pachêco-Pereira, Neelambar R. Kaipatur, June Cheung, Paul W. Major, Lawrence H. Le

**Affiliations:** 1 Department of Radiology & Diagnostic Imaging, University of Alberta, Edmonton, Alberta, Canada; 2 Department of Biomedical Engineering, University of Alberta, Edmonton, Alberta, Canada; 3 School of Dentistry, University of Alberta, Edmonton, Alberta, Canada; 4 Department of Comprehensive Dentistry, UT Health San Antonio, San Antonio, Texas, United States of America; 5 Department of Communication Sciences and Disorders, University of Alberta, Edmonton, Alberta, Canada; Chongqing University, CHINA

## Abstract

**Background and objective:**

The current methods to image alveolar bone in humans include intraoral 2D radiography and cone-beam computed tomography (CBCT). However, these methods expose the subject to ionizing radiation. Therefore, ultrasound imaging has been investigated as an alternative technique, as it is both non-invasive and free from ionizing radiation. In order to assess the validity and reliability of ultrasonography in visualizing alveolar bone, a systematic review was conducted comparing ultrasound imaging to CBCT for examination of the alveolar bone level.

**Study design:**

Seven databases were searched. Studies addressing examination of alveolar bone level via CBCT and ultrasound were selected. Risk of bias under Cochrane guidelines was used as a methodological quality assessment tool.

**Results:**

All the four included studies were *ex vivo* studies that used porcine or human cadaver samples. The alveolar bone level was measured by the distance from the alveolar bone crest to certain landmarks such as cemento-enamel junction or gingival margin. The risk of bias was found as low. The mean difference between ultrasound and CBCT measurements ranged from 0.07 mm to 0.68 mm, equivalent to 1.6% - 8.8%.

**Conclusions:**

There is currently preliminary evidence to support the use of ultrasonography as compared to CBCT for the examination of alveolar bone level. Further studies comparing ultrasound to gold standard methods would be necessary to help validate the accuracy of ultrasonography as a diagnostic technique in periodontal imaging.

## Introduction

The periodontium is a complex tooth-supporting structure consisting of four main components: the alveolar bone, cementum, gingiva, and periodontal ligament [1]. Each entity has a unique composition and distinct function as compared to the other. Alveolar bone composing of the alveolar process of the jaws and alveolar bone proper forms the tooth socket and provides attachment for periodontal ligament and tooth [2]. Alveolar bone loss is multifactorial in nature. Bone loss due to bacterial infection of teeth (dental caries), which if left untreated will result in pulpitis extending towards tooth’s apex. In this situation, a periapical abscess may form in the alveolar bone adjacent to the apex leading to bone loss [2]. Many local and systemic diseases such as osteoporosis, Papillon-Lefevre syndrome, Down syndrome, HIV infection, neutropenia, Chediak-Higashi syndrome, can also lead to bone loss in the oral cavity [3]. The two most common reasons for alveolar bone loss are periodontitis and residual ridge resorption. Bacterial infection resulting in inflammation of the periodontium (periodontitis) may result in bone loss and subsequent loss of teeth, if left untreated. Research has shown an association between periodontal disease and other medical disorders such as diabetes, cardiovascular and respiratory illnesses, as well as pre-term and low-birth-weight babies [4–7]. The deterioration of arterial stiffness and vascular endothelial function were found to be correlated with clinical attachment loss and alveolar bone loss in patients with severe periodontitis [8], which was considered as the sixth-most prevalent disease in the world [9].

A variety of clinical and radiographic methods are currently used to evaluate periodontal status [10]. For instance, periodontal probing can provide information about sulcus depth. However, some reports indicate that inflammation of the periodontium could affect probe penetration and accuracy [11, 12]. Furthermore, pocket depth measurement does not provide direct assessment of alveolar bone level. A different method is 2D intra-oral radiography, which provides information regarding alveolar bone level on the mesial and distal aspects of tooth [12], but not the bony defects on the buccal and lingual surfaces of the teeth.

Cone-beam computed tomography (CBCT) is currently used in dentistry to image hard tissues, and has been used to examine periodontal defects and bone loss in recent years [13–15]. It has advantages over the traditional radiographic imaging due to its 3D capabilities to view images. CBCT currently renders the only radiographic method to visualize the bony contour on the buccal and lingual surfaces [14]. A common way to measure alveolar bone level is to measure the distance from a reference landmark such as cemento-enamel junction (CEJ) to alveolar bone crest with or without comparing with the root length [16, 17]. Leung et al. scanned 334 teeth from human dry skulls by CBCT and found that, with a 0.38 mm reconstructed isotropic voxel, the CEJ and alveolar bone height could be measured with an accuracy of 0.4 mm and 0.6 mm respectively as compared with direct measurement by a digital caliper [18]. An *in vivo* study in human subjects found CBCT to be more accurate than intra-oral radiography in determining the morphology of vertical bone defects on the distal and mesial aspect of the tooth, when compared with gold standard direct surgical measurements [19]. Recent systematic reviews indicated that CBCT is currently the most accurate method available to determine the morphology of intra-bony defects with/without furcation involvement [13, 17]. However, these studies did not recommend CBCT as a routine use for patients because of high radiation dose and steep financial cost.

Ultrasonography uses the reflections or echoes of the ultrasound signals to image the internal structures of the tissues [20]. It offers a non-invasive method that does not expose the subject to ionizing radiation. Ultrasound is mechanical wave with frequency higher than 20 kHz. The frequency used in medical ultrasonography, mostly ranging from 2 MHz to 15 MHz [21], depends on the imaging depth and size of the structures. In B-mode imaging, a 2D greyscale image can be obtained using a linear array of transducers. B-mode ultrasound is mainly used to image soft tissues such as organs, muscles, vessels, mucosa, etc. Recently, ultrasound has been applied to study the properties of bone tissue [22, 23], to estimate the cortical bone thickness [24], and to image spine in children with scoliosis [25].

Ultrasound has been considered a promising tool for imaging hard dental structures [26], especially alveolar bone [27–30]. However, there are no systematic reviews on the validity and reliability of ultrasound imaging for periodontal bone loss in comparison with the clinical CBCT. Different from traditional review, a systematic review has a transparent protocol which helps minimize the bias in choosing and rejecting articles [31]. Therefore, the aim of this work is to perform a systematic review on the agreement of ultrasound comparing with CBCT as a diagnostic tool to image the alveolar bone level.

## Material and methods

The systematic review was carried out following the guidelines set out in the Preferred Reporting Items for Systematic reviews and Meta-Analyses (PRISMA) [32]. A detailed PRISMA checklist can be found in S1 File.

### Protocol and registration

A protocol was submitted to PROSPERO with the Centre for Reviews and Dissemination with the University of York under CRD42016038475. Information of the protocol is available at http://www.crd.york.ac.uk/prospero/display_record.asp?ID=CRD42016038475.

### Study design and eligibility criteria

Our systematic review aimed to answer a specific question which was formed following PICOS principle. The population (P) is the alveolar bone in animal and/or human. The intervention (I) is ultrasound imaging. The comparison (C) is CBCT imaging. The outcome (O) is the distance from the alveolar bone crest to a reference anatomical landmark. The types of study (S) are diagnostic imaging studies. After determining the populations and types of studies of interest, the inclusion and exclusion criteria were also settled upon. This included diagnostic studies that compared measurements acquired from 2D ultrasound images and CBCT images. There was no language restriction in database searches. Papers that used ultrasound as a tool for debridement and scaling or used the RF (radio-frequency) signals were excluded because they did not involve 2D images. Review papers and conference abstracts were not considered as eligible.

### Information sources and search

The protocol also included a list of search terms. The search phrases were combined with additional search headings that were tailored for each database as per S1 Table. These databases included CINAHL, the Cochrane Library, EMBASE, LILACS, MEDLINE, PubMed, and the Web of Science. Google Scholar was elected as the grey literature search machine. The search was conducted until December 31, 2017.

### Study selection

The search results occurred in two phases reviewed independently by three researchers (KCN, JMC, and NRK). In the first screening phase, the search results were evaluated based on their titles and abstracts. All the articles that fit the initial selection criteria had full text retrieved and reviewed independently based on inclusion or exclusion criteria, and then a final selection of articles was made. Full-text articles were hand searched for potential references that might have been missed by electronic search. Any initial disagreement between the researchers was settled through consultation with an expert in the field (LHL) and continued discussion, until a consensus was reached.

### Data collection process and data items

Cochrane handbook was used as the guideline for data collection process [33]. The data extraction followed a structured approach and a template was created to extract key features from each included paper. Two reviewers (KCN and JMC) extracted the data. A third reviewer (NRK) crosschecked and confirmed accuracy of key information. When there is a disagreement between the researchers, the experts (LHL, PM, CP-P) in the field were involved for discussion until a final decision was made.

The data item list includes first author’s name, year of publication, study design & sample size, selected target, specifics of the ultrasound and CBCT scanning devices, relevant findings & conclusions, the mean difference and/or agreement of the comparison between the two methods.

The authors would be contacted for clarification or missing raw data.

### Risk of bias in individual studies

The studies underwent a risk of bias assessment under Cochrane guidelines [33] using Review Manager 5.3, which can be accessed at http://community.cochrane.org/tools/review-production-tools/revman-5.

### Summary measures

Primary outcome will be the difference between ultrasound and CBCT measurements of the alveolar bone level, which is measured as the distance from the alveolar bone crest to a specific landmark such as CEJ or gingival margin. Secondary outcome measures the agreement and correlation between the two methods for the selected targets.

### Synthesis of results, risk of bias across studies, and additional analysis

The data from the included studies were organized and characterized. Descriptive statistical analysis using the mean and standard deviation, absolute difference, the Bland-Altman plot for agreement, and linear regression for correlation were used to evaluate the outcomes. A correlation greater than 0.8 is described as strong, a correlation from 0.5 to 0.8 is reported as moderate, and a correlation less than 0.5 is considered as weak. The agreement of ultrasound and CBCT measurements is expected to lie within the 1 mm error range commonly acceptable for direct measurement using a periodontal probe. Meta-analysis and risk of bias across study will be conducted if the data allows.

## Results

### Study selection and characteristics

**Fig 1:**
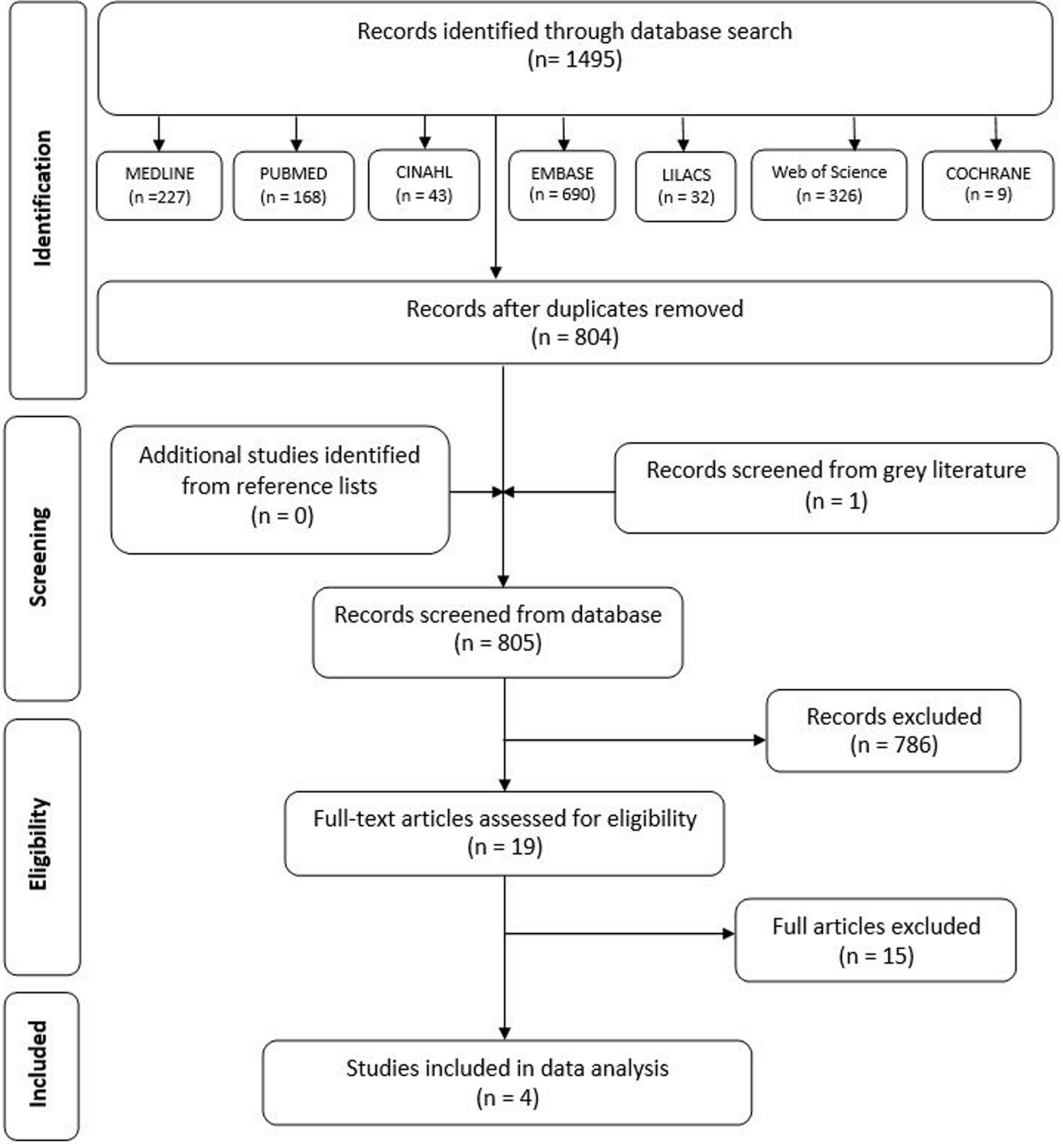
Modified PRISMA flow chart with the database search and resultant screening process [32].

Fig 1 presents the flow diagram of the results from the study selection process. The search results from seven databases produced 1495 articles. Following initial selection based on title and abstract screening, 19 articles were selected for phase 2 of the selection process. After the retrieval and reading the full text, 4 articles fulfilled all the inclusion and exclusion criteria. The reasons for exclusion were summarized in S2 Table [34–48].

The four chosen studies were published recently from 2011 to 2017 [49–52]. They were *ex vivo* studies and had small sample sizes except the most recent study by Chan et al. (2017b) that explored 144 teeth of 6 cadavers [52]. The studies used different subjects (animal carcass vs. human cadaver), different measuring positions (lingual side vs. labial side), and different parameters (CEJ to alveolar bone crest vs. gingival margin to alveolar bone crest). Different CBCT systems were used with resolution ranging from 0.08 to 0.2 mm. The ultrasound scanners had high frequency ranging from 14 MHz to 20 MHz. In the included studies, there was no sample/power calculation provided. For the studies of Nguyen et al. (2016) [50], Chan et al. (2017a) [51], and Chan et al. (2017b) [52], all the raters were calibrated. In Chifor et al. (2011), the information about the rater was not mentioned [49].

### Risk of bias within studies

**Fig 2:**
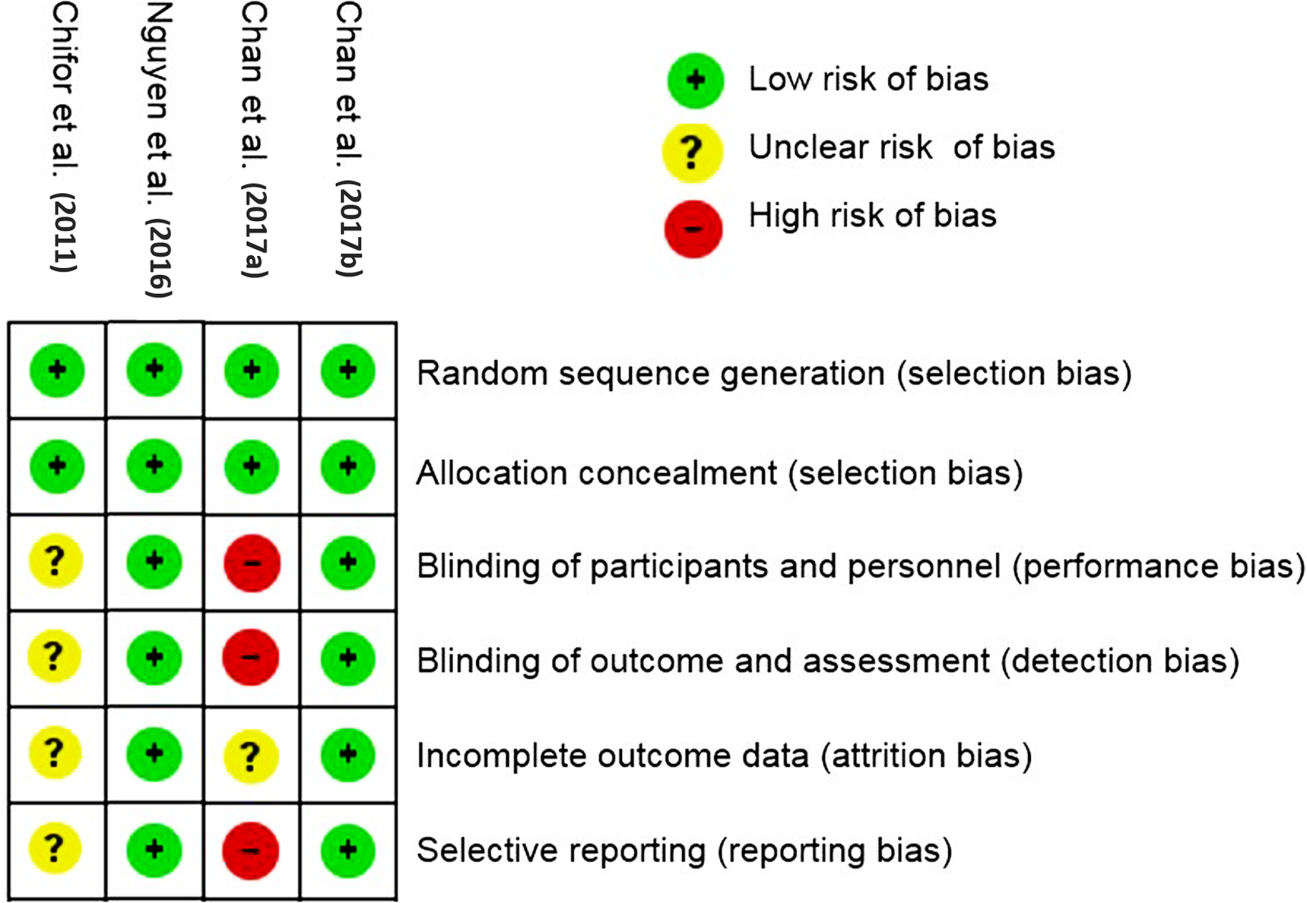
Risk of bias of the included studies.

The risk of bias assessment was shown in Fig 2. The original authors did not explicitly state if there were any steps taken to blind the examiners between measurements, which constitutes a major reason of unclear bias within the studies.

### Results of individual studies

Chifor et al. (2011) aimed to identify the reference marker to monitor horizontal bone resorption using ultrasound and measure its accuracy by comparing the measurements of ultrasound or CBCT with microscopy, which was considered a gold standard [49]. There was no information on the time delay between these two scans. They calculated alveolar bone level by measuring the distance from CEJ to alveolar bone crest.

Differently, Nguyen et al. (2016) aimed not only to image hard dental tissues and periodontal attachment apparatus using ultrasound and calculate its agreement with CBCT, but also to analyze the reflection events or echoes coming from the interfaces of the soft tissue and tooth tissues [50]. The distances from gingival margin to alveolar bone crest were measured three times by two raters with three-day intervals between measurements.

Chan et al. (2017a) investigated the use of ultrasound to image the facial bone surface and soft tissue of maxillary anterior teeth, greater palatine foramen, mental foramen, and lingual nerve [51]. The measurements of these specific vital structures were compared with those using CBCT and direct reading. In a recent study by the same group, Chan et al. (2017b) imaged by means of ultrasound different areas of the mouth including the anteriors, premolars and molars for a total of 144 teeth in both maxilla and mandible of 6 human cadavers [52]. Their goal was to evaluate the accuracy of ultrasound in measuring the facial crestal bone level and thickness in comparison with CBCT and direct measurement. Although Chan et al. (2017b) [52] used the same ultrasound and CBCT system as their previous study [51], the resolution of CBCT images was enhanced from 0.2 mm to 0.08 mm.

The image acquisition parameters and study characteristics of the included studies are presented in Table 1 [49–52].

**Table 1:** Summary of image acquisition parameters and study characteristics.

Study (year)	Subject and measured target	Ultrasound acquisition (equipment, sample size and raters)	CBCT acquisition (equipment, sample size and raters)
Chifor et al. (2011) [49]	- Lingual sides of porcine mandibular anterior specimens - Distance from CEJ to alveolar bone crest	- Ultrasound DermaScan C scanner (Cortex Technology, Hadsund, Denmark) with single element transducer at 20 MHz - 20 samples from 4 porcine mandibles (used 18 for comparison) - 1 rater measured once	- CBCT unit New Tom 3G (Verona, Italy) with 0.2 mm voxel - 20 samples from 4 porcine mandibles (used 18 for comparison) - 1 rater (same as US) measured once
Nguyen et al. (2016) [50]	- Labial sides of porcine mandibular central incisor specimens - Distance from gingival margin to alveolar bone crest	- Ultrasound SonixTablet scanner (Analogic, Vancouver, BC, Canada) with 128-element linear array transducer (L40-20/12) at 20 MHz - 2 samples from one porcine mandible - 2 raters, each measured 3 times	- CBCT i-CAT scanner (Imaging Sciences International, Hatfield, PA, USA) with 0.2 mm voxel, 120 kVp, 18.54 mAs, scan time of 20s, and 16 cm × 96 cm FOV - 2 samples from one porcine mandible - 2 raters (same as US), each measured 3 times
Chan et al. (2017a) [51]	- Labial sides of cadaver maxillary anterior specimens - Distance from CEJ to alveolar bone crest	- Ultrasound ZS3 scanner (Zonare, Mountain View CA, USA) with 128-element linear array transducer (L14-5sp) at 14 MHz - 6 samples from one cadaver head - 1 rater measured once	- CBCT 3D Accuitomo 170 scanner (JMorita, Japan) with 0.2 mm voxel, 120 kVp, 18.66 mAs, and scan time of 20 s - 6 samples from one cadaver head - 1 rater (same as US) measured once
Chan et al. (2017b) [52]	- Labial sides of cadaver anterior, premolar and molar in maxilla and mandible - Distance from CEJ to alveolar bone crest	- Ultrasound ZS3 scanner (Zonare, Mountain View CA, USA) with 128-element linear array transducer (L14-5sp) at 14 MHz - 144 samples from 6 cadaver heads (5 samples were excluded due to inadequate image quality) - 1 rater measured once	- CBCT 3D Accuitomo 170 scanner (JMorita, Japan) with 0.08 mm voxel, 120 kVp, 18.66 mAs, and scan time of 20 s - 144 samples from 6 cadaver heads - 1 rater (different from US) measured once

### Synthesis of results, risk of bias across studies, and additional analysis

Table 2 show the summarized results of the comparison between ultrasound and CBCT measurements for all four included studies. Summary of the findings included sample size, mean ± standard deviation of ultrasound (μ_*US*_ ± ơ_*US*_) and CBCT (*μ*_*CBCT*_ ± ơ_*CBCT*_), mean difference (MD) (|μ_*US*_ − μ_*CBCT*_| (*mm*) and $\frac{|\mu_{US}-\mu_{CBCT}|*100}{(\mu_{US}+\mu_{CBCT})/2}(\%)$), correlation (*R*, *p*), bias ($\frac{\sum {(US}_{i}-{CBCT}_{i})}{N}$) and 95% limit of agreement (LoA).

**Table 2:** Summarized findings for the comparison between ultrasound and CBCT measurements. The acronym “US” refers to ultrasound.

Study (year)	Measured outcome	*N*	μ_*US*_ ± ơ_*US*_ (mm)	μ_*CBCT*_ ± ơ_*CBCT*_ (mm)	MD (mm) (%)	Correlation (*R*, p*)	Bias (US-CBCT)	95% LoA (mm)
Chifor et al. (2011) [49]	Distance from CEJ to alveolar bone crest	18	4.37 ± 2.16	4.45 ± 2.07	0.07 (~1.6%)	0.98, *p* < 0.01	-0.07	[-0.97,0.83]
Nguyen et al. (2016) [50]	Distance from gingival margin to alveolar bone crest	2	7.37 ± 0.15	8.05 ± 0.18	0.68 (~8.8%)	N/A	N/A	N/A
Chan et al. (2017a) [51]	Distance from CEJ to alveolar bone crest	6	4.3 ± 1.1	4.6 ± 0.4	0.3 (~6.7%)	N/A	N/A	N/A
Chan et al. (2017b) [52]	Distance from CEJ to alveolar bone crest	138	2.66 ± 0.86	2.51 ± 0.82	0.15 (~5.8%)	0.78, *p* < 0.001	0.09	[-1.00,1.18]

* If *p* < 0.05, the correlation coefficient is considered statistically significant.

**Fig 3:**
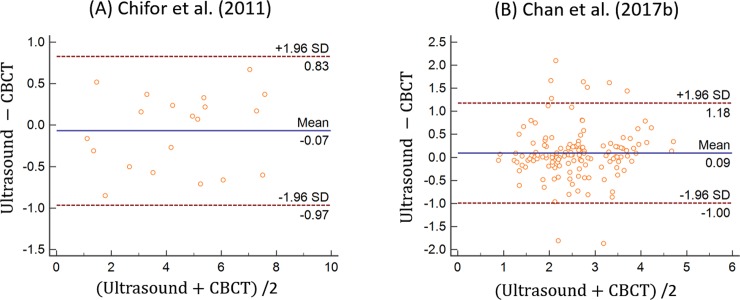
Agreement between ultrasound and CBCT in Chifor et al. (2011) and Chan et al. (2017b) using Bland-Altman plotting.

It is noted that Chifor et al. (2011) did not directly compare ultrasound images with CBCT [49]. From the data published in the paper, we were able to extrapolate the results by carrying out various statistical comparisons of the two different techniques. The difference in means or the bias between ultrasound and CBCT was smaller than 0.1 mm with the 95% limit of agreement from -0.97 mm to 0.83 mm (Fig 3A). The results showed a strong positive correlation between ultrasound and CBCT (*R* = 0.98, *p* < 0.01), higher than the reported correlation between ultrasound and microscopy (*R* = 0.79, *p* < 0.0001).

In the work by Nguyen et al. (2016), the mean values of the gingival margin—alveolar bone crest distance by ultrasound were 7.40 ± 0.23 mm for rater 1 and 7.33 ± 0.07 mm for rater 2 respectively [50]. For CBCT, the reported mean values were 8.01 ± 0.27 mm for rater 1 and 8.08 ± 0.07 mm for rater 2. On average, the absolute difference between ultrasound and CBCT measurements in measuring the alveolar bone level from gingival margin was 0.68 mm (8.8%), with CBCT estimates larger than the ultrasound measurements. The correlation and mean absolute difference were not reliable due to the small sample size (*N* = 2).

In the study of Chan et al. (2017a), the mean values of the distance between alveolar bone crest and CEJ were reported at 4.3 ± 1.1 mm, 4.6 ± 0.4 mm, 4.1 ± 0.9 mm for US, CBCT, and direct measurement, respectively [51]. Among the three methods, CBCT’s mean value was the largest, over the gold standard direct measurement by 0.5 mm and ultrasound by 0.3 mm. Although ultrasound had mean value closer to the gold standard than CBCT, its variation (standard deviation) was larger than that of CBCT. The correlation and bias could not be calculated due to the small sample size (*N* = 6).

In a recent study also by the Chan’s group [52], the means of alveolar bone level measured by ultrasound, CBCT, and direct measurements for cadaver samples were 2.66 ± 0.86 mm, 2.51 ± 0.82 mm, and 2.71 ± 1.04 mm, respectively. The correlation between ultrasound and CBCT measurements was reported at 0.78, smaller than the correlation between ultrasound and direct measurement (*R* = 0.88). The bias between ultrasound and CBCT/direct measurement was reported at 0.09 mm. However the 95% limit of agreement between ultrasound and CBCT (-1.00 to 1.18 mm) (Fig 3B) was wider than that between ultrasound and direct measurement (-0.98 to 0.8 mm) [52].

The three studies used different models (porcine models and human cadaver), different measured positions (lingual side vs. labial side), and different parameters (CEJ to alveolar bone crest vs. gingival margin to alveolar bone crest). Because the subject types in the studies were heterogeneous, there was a risk of bias across studies and hence a meta-analysis could not be performed.

## Discussion

CBCT is a relatively new imaging tool to assess alveolar bone loss three-dimensionally. CBCT images are acquired using cone-beam X-rays with a 2D matrix of detectors (flat-panel detectors). The advantage of 3D volume dataset allows periodontists and orthodontists to visualize the alveolar structures on the lingual and buccal sides, which cannot be visualized on a 2D radiograph. Currently, the use of CBCT is commonly restricted to the assessment of the hard tissues such as bone, tooth, implant or dry skull due to poor soft tissue contrast [53, 54].

On the contrary, ultrasound is an ionizing radiation-free, non-invasive imaging modality, and its application in dentistry, especially in periodontics, has been investigated since early 1963 [55]. Although ultrasound has been available in dentistry for a long time, its use in day-to-day clinical practice has not been established. The goal of this systematic review was to estimate the difference and agreement between ultrasound and the current clinical standard CBCT to determine if ultrasound can be a viable supplement to CBCT for periodontal diagnostic imaging.

All included studies were *ex vivo* investigations performed on human cadavers and porcine carcasses. Chan et al. (2017a) was a pilot study with very small sample size [51]. However, the resolution of CBCT images and the cadaver samples used in Chan et al. (2017a) were different from the one in Chan et al. (2017b) [51, 52]. Therefore, we considered them to be two different studies. The data gathered by our study supported a strong agreement and correlation between the measurements taken using CBCT and ultrasound imaging. The absolute difference between ultrasound and CBCT varied from 0.071 mm to 0.68 mm (1.6% to 8.8%), where ultrasound measurements consistently underestimated CBCT measurements. In other words, ultrasound showed the bone level higher relative to CEJ than CBCT. However, this raised a question whether CBCT would overestimate the true value as reported [15, 51]. The low level of evidence and high risk of bias were also presented in a systematic review on the accuracy of CBCT in assessing periodontal defects from 2000 to 2015 [56]. The study found fourteen articles with the mean errors of CBCT ranging from 0.19 ± 0.11 mm to 1.27 ± 1.43 mm in comparison with direct measurement.

The investigations by Chifor et al. (2011) and Chan et al. (2017a) have shown small difference and high correlation between ultrasound and the gold standard methods (microscopy or direct measurements) in measuring the distance from CEJ to alveolar bone crest. Besides measuring distances between vital structures such as bone crest and CEJ, the two aforementioned studies also concluded that ultrasound could be used to image periodontium and hard tissue surfaces [49, 51]. Their conclusions are in agreement with Tsiolis et al. [29], who compared ultrasound with direct measurement in locating alveolar bone crest. A limitation in the two studies was that there was only one rater to measure the data once. This could probably create a bias due to human error and random error, which may result in greater or lesser consistency in locating the bone crest or CEJ.

The study by Nguyen et al. (34) was the first report that presented high quality ultrasound images of the tooth-periodontium and used a combination of ultrasound physics, travel-time computation, and wave field simulation to interpret the results. The study concluded that ultrasound had high potential to be an ionizing radiation-free and non-invasive diagnostic imaging tool for assessing tooth-periodontium structures. Although there were two raters who did the measurement three times, further tests should be performed to confirm the results presented, as the sample size was small.

The difference in ultrasound systems would also affect the result for alveolar bone imaging. The lateral resolution is best at the focal depth or the near field distance, which can be determined by (transducer diameter)^2^ × frequency /(4 × velocity) for unfocused transducer [20]. Chifor et al. used a 20 MHz single element transducer, which had a single focal depth and thus would not provide image with the best lateral resolution and image quality [49]. Chan’s group used a 14 MHz phased array transducer [51, 52]. Each A-beam was generated by a group of elements, which were electronically steered to image at multiple focal depths with extended lateral resolution. The lower frequency allowed more depth of penetration but lower resolution. Nguyen et al. used a 20 MHz linear array transducer with 128 elements of 0.1 mm pitch (element-to-element separation), which significantly enhances the signal-to-noise ratio, resolution, and image quality [50]. However, the difference between ultrasound and CBCT reported in Nguyen’s study was higher than the other two included studies. The reason may come from the poor image contrast of the gingival margin by CBCT.

Medical ultrasound imaging relies on the echoes coming from the tissue interfaces. The echo arises due to the presence of impedance contrast between different tissues and its strength depends on the magnitude of the impedance contrast. Choi et al. (2012) used a sector scanning ultrasound probe (a single transducer with a rotor) to image porcine model for implant studies and demonstrated that bone surfaces and implants were visibly identified as strong reflectors in the images including the breaks on cortical layer in the nerve canal [42]. The alveolar bone is composed mostly of cancellous bone and a very thin cortical plate. Assuming that the impedances of gingiva and cortical bone are 1.63 MRayl [50] and 7.38 MRayl [57] respectively, the echoes, which are visibly identified on the ultrasound images, carry about 41% of the incident energy and delineate a good estimation of the thickness of the gingiva. The amount of reflected energy was calculated without considering the attenuation of gel pad and the transmission loss across the gel pad interfaces. The interaction of ultrasound with cancellous bone involves multiple scattering within the porous alveolar bone. The surface of alveolar bone is rough and thus, non-specular reflection occurs with energy scattering in all directions. The echoes from the interface is not well focused and the subsequent imaged interface is not well defined but appears as a zone [50]. Even though in theory there is about 59% incident energy transmitted across the gingiva—cortical bone interface into the alveolar bone, the actual incident energy striking the bottom of the alveolar layer is weak due to scattering and attenuation of alveolar bones. Therefore, the corresponding echoes are less likely to be detected. For this reason, the thickness of the alveolar bone could not be determined, except at the crestal bone and the same phenomenon was observed by others [28, 49, 50].

### Implications for clinical practice

CBCT has immensely helped clinicians further evidence-based treatment, which was not possible with 2D radiography. The accuracy of CBCT depends on the scanning voxel size and the field of view (FOV), which in turn depends on the imaged region of interest (ROI). Large FOV results in increased patient radiation dose and scattered radiation, which enhances image noise, and degrades contrast and image sharpness especially for soft tissues [58].

The optimum FOV should include the entire ROI but always be as small as possible for better image quality with less scatter and lower radiation. The small voxel size will provide high image resolution but also enhanced radiation exposure. As children and adolescents are extremely sensitive to increased radiation, it is crucial for practitioners to adhere to the ALARA (as low as reasonably achievable) principle [59]. In fact, CBCT imaging delivers much higher radiation dose than conventional intraoral radiography, about 5–74 times that of a single film-based panoramic radiograph [53]. Children are increasingly susceptible to deleterious effects of ionizing radiation due to faster rate of cellular growth, organ development, and longer life expectancies. Children’s susceptibility to ionizing radiation is based on stochastic risk of effective dose [60]. This effect can lead to irreversible alteration in cells, presumably by damaging cellular DNA. There is no safe limit for ionizing radiation exposure in oral diagnostic imaging. The accumulation of radiation dose from CBCT due to repeated visits could have harmful effects on the subject [61]. This has prompted the American Academy of Oral and Maxillofacial radiology to issue, in their position statement, the strict guidelines for the use of CBCT [62]. Development of high quality imaging modalities that do not expose patients to ionizing radiation is very important. On average, a large FOV CBCT scan takes about 20–30 s for all teeth. But the dentist need to wait for about 5 min for the image reconstruction to complete prior to viewing. For the 2D ultrasound imaging, it may take about 15 s for a scan (one tooth). However, the dentist can view the structures in ultrasound images in real time (during the scan).

The results of this review demonstrate that ultrasound has the potential to be an ionizing radiation-free imaging modality to visualize the alveolar bone contour on the buccal and lingual surfaces. Beside assessing the alveolar bone level, the use of ultrasound in diagnosing periapical inflammatory lesions of the jaw and periodontal bony defect has also been demonstrated [63, 64]. High frequency ultrasound imaging (40 MHz) can also help accurately measure the gingival thickness and probing depth [65]. Especially in radiolucent areas where the cortices are thinned or absent, ultrasound imaging is highly possible for the reasons described previously. For children whose alveolar processes are thinner, the use of ultrasound to detect the internal structures such as the presence of a developing tooth at different growth stages is another possibility, which needs further investigation. This would strengthen the value of ultrasound for use in children and adolescents to provide better, safer, and ionizing radiation-free oral care.

### Implications for research

CBCT is a relatively new tool for periodontal bone examination and is not considered a gold standard while its own accuracy and validity have been under examination. A direction for further research should include comparing ultrasound imaging to direct measurement, which is currently taken as the “gold standard” for some aspects of periodontal assessment. This could provide a better comparison, even though there are inherit deficiencies associated with direct measurement, such as less sensitive tools that are calibrated to record in millimeters as well as variation between examiners.

Ultrasonography uses the echoes of mechanical waves to image the internal dento-periodontal structures. The generation and reception of mechanical vibrations can be accomplished by the ultrasound transducers. High frequency ultrasound generates signals of smaller wavelength, which can be used to study small-scale structures. However, high frequency signals cannot propagate greater depth due to intrinsic absorption of the tissue. A study using a range of frequencies should be investigated to find an ideal frequency for oral applications.

Deciding a proper sample size is also an important step during any study design to enhance power and reduce estimation error. In animal study, the number of samples approved for a scientific experiment should be justified. It will save time and resources by acquiring enough samples to achieve the required expectation. The method to calculate the sample size varies for different experimental design and assumption. The estimated sample size, *N*_*s*_ required for comparing the means between two methods A and B using a two-sided test with significant level *α* and power (1 − *β*) is given by $N_{s}=\frac{{(ơ}_{A}^{2}+{ơ}_{B}^{2}){\left(z_{1-\frac{\alpha }{2}}+z_{1-\beta }\right)}^{2}}{{\left|\mu_{A}-\mu_{B}\right|}^{2}}$ where μ and σ denote the mean and standard deviation, and z_*x*_ refers to the upper x’th quantile of the standard normal distribution [66]. Normally the power may be selected as 80% (1 − *β* = 0.8) and the significant level or risk of Type I error (*α*) as 5% or 10%. The mean and standard deviation of the measured population may be attained from a pilot study or literature. The sample size will increase when the standard deviations of the two measured groups are large or when the difference between two groups is small. For example, Chifor et al. (2011) and Chan et. al. (2017a) had similar mean values but the sample size acquired for Chan et al.’s study (2017a) was only 69 samples while the sample size for Chifor et al.’s study (2011) was up to 6301 samples to meet the expected power of 80% and significant level of 10%.

### Limitations

Caution should be exercised in drawing conclusions regarding the accuracy and validity of ultrasound imaging as a diagnostic tool for the alveolar bone, because of a very small number of studies included in this systematic review and the lack of statistical power within these studies. Further studies with sufficiently large sample size, would be required to verify these preliminary results. In addition, as all the included studies were *ex vivo* in nature, future studies should be conducted on human subjects.

## Conclusion

The results of this systematic review are limited due to the sample size of studies and are associated with unclear risk of bias, as all the studies were *ex vivo* in nature. *In vivo* studies comparing ultrasound to other methods with sufficiently large sample size would be necessary to help validate the accuracy of ultrasonography as a diagnostic technique in dentistry.

## Supporting information

S1 File PRISMA 2009 checklist. (DOC)

S1 Table Databases and individualized truncations of key words. (DOCX)

S2 Table Excluded papers and reasons. (DOCX)
