# Supplementary material for: Comparison of ultrasound imaging and cone-beam computed tomography for examination of the alveolar bone level: A systematic review
Source: PLoS One. 2018 Oct 3;13(10):e0200596. doi: 10.1371/journal.pone.0200596 (PMC6169851; doi:10.1371/journal.pone.0200596)
Supplement: S1 Table — (DOCX) [file pone.0200596.s002.docx]

**S1 Table. Databases and individualized truncations of words**

| **Database** | **Key words and truncation** |
| --- | --- |
| MEDLINE (n= 227)  https://web.library.ualberta.ca/databases_help/ovid_medline/index.cfm  &  EMBASE (n=690)  https://www.embase.com/search | ((alveolar bon*.mp.) OR (alveolar bone level) OR (cementoenamel junction*.mp.) OR (cemento-enamel junction*.mp.) OR (dehiscence*.mp.) OR (fenestration*.mp.) OR (periodont*.mp.) OR (exp alveolar bone loss/) OR (exp periodontium/)) AND ((ultrasonic*.mp.) OR (ultrasonogra*.mp.) OR (ultrasound*.mp.) OR (exp echography/)) AND ((CBCT.mp.) OR (compute* tomography.mp.) OR (conebeam.mp.) OR (cone-beam.mp.) OR (tomogra*.mp.) OR (exp cone beam computed tomography/)) |
| PubMed (n=168)  https://www.ncbi.nlm.nih.gov/pubmed | ((“alveolar bone“) OR (“alveolar bone level“) OR (“cementoenamel junction“) OR (“cementoenamel junctions“) OR (“cemento-enamel junction“) OR (“cemento-enamel junctions“) OR (“dehiscence”) OR (“fenestration”) OR (periodont*) OR (“periodontium” OR (“alveolar bone loss”))) AND ((“ultrasonic”) OR (“ultrasonics”) OR (“ultrasonography”) OR (“ultrasound”)) AND ((CBCT) OR (“cone beam“) OR (“cone-beam”) OR (tomogra*) OR (“cone beam computed tomography“)) |
| CINAHL (n=43) https://health.ebsco.com/products/the-cinahl-database | ((alveolar bon*) OR (alveolar bone level) OR (cementoenamel junction*) OR (cemento-enamel junction*) OR (dehiscence*) OR (fenestration*) OR (periodont*) OR (MH “Cementoenamel Junction”) OR (MH “Periodontium+”)) AND ((ultrasonic*) OR (ultrasonogra*) OR (ultrasound*) OR (MH “Ultrasonography+”)) AND ((CBCT) OR (compute*tomography) OR (conebeam) OR (cone-beam) OR (tomogra*)) |
| LILACS (n=32)  lilacs.bvsalud.org/en/ | tw:(alveolar bone* OR cementoenamel junction* OR cemento-enamel junction* OR dehiscence* OR fenestration* OR periodont* OR mh:"periodontium") tw:(ultrasonic* OR ultrasonogra* OR ultrasound* OR mh:"ultrasonography") tw:(cbct OR compute* tomography OR conebeam OR cone-beam OR tomogra* OR mh:"cone beam computed tomography") |
| Web of Science (n=326)  http://apps.webofknowledge.com | ((alveolar bon*) OR (alveolar bone level) OR (cementoenamel junction*) OR (cemento-enamel junction*) OR (dehiscence*) OR (fenestration*) OR (periodont*)) AND ((ultrasonic*) OR (ultrasonogra*) OR (ultrasound*)) AND ((CBCT) OR (compute*tomography) OR (conebeam) OR (cone-beam) OR (tomogra*)) |
| Cochrane Library (n=9)  www.cochranelibrary.com | ((alveolar bon*:ti,ab,kw) OR (cementoenamel junction*:ti,ab,kw) OR (cemento-enamel junction*:ti,ab,kw) OR (dehiscence*:ti,ab,kw) OR (fenestration*:ti,ab,kw) OR (periodont*:ti,ab,kw) OR (MeSH descriptor: [Alveolar Bone Loss] explode all trees) OR (MeSH descriptor: [Periodontium] explode all trees)) AND ((ultrasonic*:ti,ab,kw) OR (ultrasonogra*:ti,ab,kw) OR (ultrasound*:ti,ab,kw) OR (MeSH descriptor: [Ultrasonography] explode all trees)) AND ((CBCT:ti,ab,kw) OR (compute* tomography:ti,ab,kw) OR (conebeam:ti,ab,kw) OR (cone-beam:ti,ab,kw) OR (tomogra*:ti,ab,kw) OR (MeSH descriptor [Cone-Beam Computed Tomography] explode all trees)) |
