# Supplementary material for: Comparison of ultrasound imaging and cone-beam computed tomography for examination of the alveolar bone level: A systematic review
Source: PLoS One. 2018 Oct 3;13(10):e0200596. doi: 10.1371/journal.pone.0200596 (PMC6169851; doi:10.1371/journal.pone.0200596)
Supplement: S2 Table — (DOCX) [file pone.0200596.s003.docx]

**S2 Table. Excluded papers and reasons**

|  | **Author, Year** | **Reason of exclusion** |
| --- | --- | --- |
| 1 | Sawada et al., 1984 [[34](#_ENREF_34)] | No comparison with CBCT |
| 2 | Todescan et al., 1994 [[35](#_ENREF_35)] | Review paper |
| 3 | Hamano et al., 2003 [[36](#_ENREF_36)] | No comparison with CBCT |
| 4 | Mazza et al., 2006 [[37](#_ENREF_37)] | No quantitative measurements  Spiral CT was compared instead of CBCT |
| 5 | Klein et al., 2008 [[38](#_ENREF_38)] | No comparison with CBCT  The velocity instead of distance was measured |
| 6 | Machtei et al., 2010 [[39](#_ENREF_39)] | No comparison with CBCT  No measurement of the alveolar bone level |
| 7 | Merheb et al., 2010 [[40](#_ENREF_40)] | No comparison with ultrasound |
| 8 | Kaneko et al., 2011 [[41](#_ENREF_41)] | Ultrasonic irrigation instead of imaging |
| 9 | Choi et al., 2012 [[42](#_ENREF_42)] | No quantitative measurements |
| 10 | Szopinski and Regulski, 2013 [[43](#_ENREF_43)] | No quantitative measurements |
| 11 | Zigdon‐Giladi et al., 2015 [[44](#_ENREF_44)] | No measurement of the alveolar bone level |
| 12 | Guo et al., 2015 [[45](#_ENREF_45)] | No comparison with ultrasound |
| 13 | Chifor et al., 2015a [[46](#_ENREF_46)] | No comparison with CBCT |
| 14 | Chifor et al., 2015b [[47](#_ENREF_47)] | No comparison with CBCT |
| 15 | Pascual et al., 2017 [[48](#_ENREF_48)] | No comparison with ultrasound |
